# Prevalence and associated factors for COPD and pre-COPD in Guizhou Province, China: A cross-sectional study

**DOI:** 10.18332/tid/215242

**Published:** 2026-03-06

**Authors:** Songjun Shao, Silu Hu, Cheng Zhang, Shan S. Rao, Guohang Yuan, Xiaoju Tang, Xianwei Ye

**Affiliations:** 1 Department of Respiratory and Critical Care Medicine, Clinical Research Center for Respiratory Disease, West China Hospital, Sichuan University, Chengdu, China; 2 Department of Respiratory and Critical Care Medicine, Guizhou Provincial People's Hospital, Guiyang, China

**Keywords:** COPD, Pre-COPD, prevalence, associated factors, epidemiological investigation

## Abstract

**INTRODUCTION:**

In China, the incidence of chronic obstructive pulmonary disease (COPD) is escalating swiftly, encompassing almost 100 million patients documented in 2018. Nevertheless, a considerable proportion of individuals persist in a Pre-COPD condition and have not been given a COPD diagnosis. Currently, the prevalence of COPD and Pre-COPD, linked to various associated factors in Guizhou Province, China, is still uncertain.

**METHODS:**

Adults aged ≥20 years were enrolled in a 2012 cross-sectional survey of Guizhou Province via multistage random sampling. All participants underwent post-bronchodilator pulmonary function tests (PFTs). The 2012 Global Initiative for COPD was used for diagnosis.

**RESULTS:**

The participants were 6400, recruited in Guizhou Province, of whom 5395 provided reliable post-bronchodilator results and were included in the final analysis. Among individuals aged ≥20 years, the overall prevalence of COPD was 9.60%, and of Pre-COPD was 8.92%. The important factors associated with COPD and Pre-COPD included smoking, household smoke pollution, and low level of education. This survey revealed that 31.96% of participants had a history of smoking, and 3.30% had successfully quit smoking. Indoor smoke exposure from biomass burning contributed to 3.24% of the total biomass. Additionally, a lower level of education was identified as a significant associated factor for COPD and Pre-COPD.

**CONCLUSIONS:**

In Guizhou, COPD and Pre-COPD prevalence are greater than those in the general Chinese population. The primary associated factors for COPD and Pre-COPD include smoking (especially mother smoking during pregnancy), exposure to kitchen cooking fumes, and low level education.

## INTRODUCTION

Chronic obstructive pulmonary disease (COPD) poses a significant global health challenge due to its elevated prevalence^1^. In 2017, it was estimated that 544.9 million individuals were affected by chronic respiratory conditions, with approximately 55% of these cases linked to COPD^2^. The World Health Organization (WHO) anticipates that by 2030, COPD will rank as the third leading cause of mortality globally^3^. In 2008, COPD was known as the fourth and third leading cause of death in urban and rural areas in China, respectively^4^. Zhong et al.^5^ conducted a survey involving 20245 adults across seven regions in China, revealing that the prevalence of COPD in individuals aged ≥40 years reached as high as 8.20%. Thus, the China Pulmonary Health study (2012–2015) spearheaded by Wang et al.^6^ indicated that the prevalence of COPD in Chinese adults aged ≥20 years was 8.60%, with the prevalence in those aged ≥40 years soaring to 13.70%. The estimated patient population in China approaches 100 million, roughly one-third of the global total, indicating that COPD incidence in China continues to rise^6^. However, the anticipated rise in COPD incidence is not surprising. Because some people with abnormal lung function trajectories but not yet meeting the diagnostic criteria may be overlooked. Although this group of people may not have obvious respiratory symptoms at present, they have a relatively high risk of developing COPD in the future. Thus, a substantial segment of the population exists in a Pre-COPD state^7^. As yet, there is no worldwide agreement on how to define early COPD. Pre-COPD simply describes people who have no chronic respiratory symptoms and structural but functional lung changes, whose post-bronchodilator FEV1/FVC is still ≥0.7 and FEV1<80%, who are at high risk of developing COPD later^7,8^.

The Global Initiative for Chronic Obstructive Lung Disease 2023 Report highlighted that COPD is a prevalent, preventable, and treatable condition, but significant underdiagnosis and diagnostic delay result in patients receiving either no treatment or inappropriate treatment^9^. To alleviate the burden of COPD, enhanced therapeutic strategies and targeted public-health interventions – together with individualized exposure-reduction measures – will be required^10^. Cigarette smoking is the single most important aetiologic factor in the development and progression of COPD: approximately 80–90% of affected individuals have a significant smoking history, and the lifetime risk of airflow limitation in persistent smokers is 3- to 5-fold higher than in never smokers^11^. It is crucial to acknowledge that environmental factors beyond tobacco smoking may also contribute to COPD and Pre-COPD. Although the hazards of the early stage of COPD are clear, there is still a lack of certainty regarding the definition and standards of ‘early COPD’, and whether intervention for patients with Pre-COPD can bring about long-term benefits. Currently, population-based data on Pre-COPD remain scarce. To address this issue, we conducted a large-scale cross-sectional survey in Guizhou Province, China.

## METHODS

### Study design and participants

**Figure 1:**
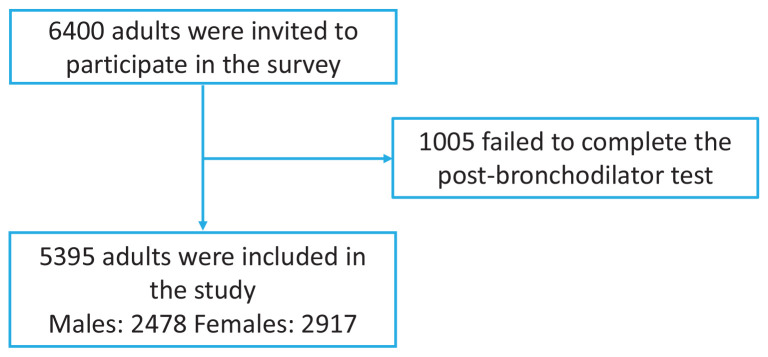
Flow chart of participants throughout the study

The study was conducted from June 2012 to May 2015. We employed simple random sampling to select participants from the research area. In Guizhou Province, we evaluated 6400 individuals. Of these, 5395 adults aged ≥20 years who had resided at their current address for a minimum of six months prior to the survey, were invited to take part (Figure 1). We excluded people who could not be interviewed or who declined to give informed consent. Additionally, we excluded individuals that were ineligible for spirometry due to various conditions: 1) chest, abdominal, or eye surgery; 2) retinal detachment; 3) a myocardial infarction within the last three months; 4) hospitalization for cardiac issues in the preceding month; and 5) pregnancy or breastfeeding. This study protocol received approval from the Ethics Review Committee of Guizhou Provincial People’s Hospital. We obtained written informed consent from all participants.

The present study was designed as a retrospective cohort analysis. Therefore, the sample size was not determined by a formal *a priori* power calculation. Instead, it was defined by the total number of individuals who met the predefined inclusion and exclusion criteria within the specified study period. All eligible participants available in the database during this period were included in the analysis to minimize selection bias and to ensure the study sample accurately reflects the real-world population.

### Procedures

Researchers attended two days of training before administering a questionnaire on demographics, COPD-related factors, medical history, and parental respiratory history. Regarding our definitions, current smokers were those who had smoked at least 100 cigarettes in their lifetime and still smoked. Passive smokers were individuals who reported inhaling other people’s smoke. Smoking during pregnancy was recorded if a mother had a history of smoking or passive smoking during pregnancy. The use of domestic fuels such as biofuels, coal, and gas was considered if it lasted longer than six months. Samples were grouped into smoke exposure (coal, biofuel) and non-exposure (electricity, gas) categories. Trained personnel assessed indoor environments for ventilation quality and kitchen/bedroom layouts. Biomass use included woody fuels or animal waste for cooking within the past six months. After the questionnaire, trained researchers conducted pulmonary function tests using a spirometer, measuring FVC and FEV1 before and after administering a bronchodilator. COPD was defined as post-bronchodilator FEV1/FVC<70%, and Pre-COPD was defined as FEV1/FVC ≥70% and FEV1 <80%, following GOLD guidelines^7^. An expert panel supervised quality control by excluding poor-quality tests.

### Statistical methods

Statistical analyses were performed using IBM SPSS Statistics (version 23.00) and R. Comparisons of continuous variables were performed using Welch’s two-sample t-test (unequal variances). Comparisons of categorical variables used the Pearson chi-squared test without continuity correction. For binomial proportion estimates, 95% confidence intervals were calculated using the Wilson method, given its superior performance for proportions. For sample means, 95% confidence intervals were computed using t-distribution-based intervals. Multiple comparisons were adjusted using the Benjamini–Hochberg false discovery rate (FDR) procedure. To ensure proper control of Type I error, FDR correction was applied across the entire set of comparisons. A p<0.05 was considered statistically significant. All hypothesis tests in the study were two-sided.

## RESULTS

### Demographic characteristics

Study participants varied by sex, age group, city or country, education level, ethnicity, occupation, body mass index, smoking history, biofuel exposure, kitchen room separation, and medical history. Overall, data from 5395 participants were included and are shown in Figure 1. There were 2478 males and 2917 females, aged 20–86 years, with an average age of 45.9 ± 13.95 years. Urban residents (4221 participants) made up 78.24% (95% CI: 77.11–79.32, Wilson method unless otherwise stated), with the remaining living in rural areas. Most participants were of Han ethnicity, with 4926 people, representing 91.31% (95% CI: 90.53–92.03); however, 469 individuals belonged to various ethnic minorities, accounting for 8.69% (95% CI: 7.97–9.47). Remarkably, 1348 people had only completed primary school, making up 24.99% (95% CI: 23.85–26.16). A total of 1777 people had completed junior high school (mean: 32.94%; 95% CI: 31.70–34.20), and 1314 had a senior high school education (mean: 24.36%; 95% CI: 23.23–25.52). Only 956 participants had attained university education or higher, accounting for 17.7% (95% CI: 16.72–18.76) of the sample. The majority of tobacco users smoked products such as cigarettes and chillums (a straight conical pipe with an end-to-end channel used for smoking). We examined 3671 people who had never smoked, representing 68.04% (95% CI: 66.79–69.28) of the sample. Others had a history of smoking, including 1546 current smokers (28.66%; 95% CI: 27.47–29.85) and 178 people who had quit smoking (3.30%; 95% CI: 2.85–3.81). Our study found that 96.1% (95% CI: 95.56–96.59) of the population had not been exposed to biofuels, such as coal, LP, gas, and grass, used for at least six months. Exposure to indoor smoke from burning biomass was 3.24% (firewood 3.26%, manure 0.06%, and coal 28.67% for more than half a year). Frying and roasting accounted for 67.04%. A total of 338 people did not have a separate kitchen from the bedroom, representing 6.27% (95% CI: 5.65–6.94) of the sample. COPD patients had a related medical history, with 216 having a history of chronic bronchitis, accounting for 4.00% (95% CI: 3.51–4.56) of all COPD cases (Table 1).

**Table 1:** Baseline demographic and exposure characteristics of adults, aged ≥20 years, in the Guizhou Province COPD Study, 2012–2015 (N=5395)

*Characteristics*	*n*	*%*
**Gender**		
Male	2478	45.90
Female	2917	54.10
**Age** (years)		
20–40	2042	37.85
Mean (95% CI) p	31.66 (31.42–31.91)	<0.0001
41–60	2449	45.39
Mean (95% CI) p	49.16 (48.95–49.38)	<0.0001
≥61	904	16.76
Mean (95% CI) p	69.16 (68.81–69.50)	<0.0001
**Residence**		
Urban	4221	78.24
Rural	1174	21.76
**Education level**		
Uneducated or elementary	1348	25.00
Middle or high school	3091	57.30
University or higher	956	17.70
**Ethnicity**		
Ethnic Han	4926	91.31
Ethnic minorities	469	8.69
**Body mass index** (kg/m^2^)		
<18.5 (underweight)	341	6.32
Mean (95% CI) p	17.58 (17.49–17.67)	<0.0001
18.5–23.9 (normal weight)	2950	54.68
Mean (95% CI) p	21.51 (21.46–21.56)	<0.0001
≥24.0 (overweight)	2104	39.00
Mean (95% CI) p	26.71 (26.61–26.82)	<0.0001
**Smoking status**		
Never smoker	3671	68.04
Current smoker	1546	28.66
Quit	178	3.30
**Exposure to biofuel/non-biofuel**		
Yes (biofuel)	210	3.90
No (non-biofuel)	5185	96.10
Fry/stir-fry/roast	3617	67.04
Coal >6 months	1547	28.67
LPG >6 months	1086	20.13
Gas >6 months	1004	18.61
Grass >6 months	176	3.26
**Kitchen separate**		
Yes	5057	93.80
No	338	6.20
**Medical history**		
Asthma	89	1.65
Chronic bronchitis	216	4.00
Emphysema COPD	103	1.91
Pulmonary heart disease	39	0.72
Bronchiectasis	8	0.15
Lung TB	66	1.22
Lung CA	65	1.20

LPG: liquefied petroleum gas. TB: tuberculosis. CA: cancer.

### Factors associated with COPD and Pre-COPD

Overall, 518 participants aged ≥20 years were diagnosed with COPD, with a prevalence of 9.60% (95% CI: 8.84–10.42, p<0.0005). Of these COPD patients, 5.17% (95% CI: 4.61–5.79) were men, and 4.40% (95% CI: 3.91–5.01) were women. The age-standardized prevalence rates of COPD across disease stages were 45.95% (95% CI: 41.70–50.25) for stage I, 45.60% (95% CI: 41.32–49.87) for stage II, 7.52% (95% CI: 5.56–10.13) for stage III, and 0.97% (95% CI: 0.41–2.24) for stage IV (Table 2). Additionally, 1264 participants were considered to have Pre-COPD, accounting for 23.43% (95% CI: 22.32–24.58) of the total subjects. When grouped by age category, according to PFT results, 518 individuals were considered to have COPD: 89, aged 20–40; 225, aged 40–60 years, and 204, ≥61 years. The prevalence of COPD increased with age in our study. The smoking rate of the investigated population was 31.96% (95% CI: 30.72–33.21), but that of COPD patients was 38.90%. The prevalence of COPD was significantly different between smokers and non-smokers (p<0.0001). However, there were 33 COPD patients whose mothers smoked during pregnancy and 217 whose mothers were passive smokers. Other risk factors, such as frequent frying, stir-fry, roasting, and cooking, were associated with COPD (p<00009). When the subjects were divided into groups according to education level, there was a significant difference (p<0.002), and the result indicated that the prevalence of COPD decreased with increasing education level. However, the prevalence of some COPD patients with an underlying history, such as asthma, chronic bronchitis, emphysema-COPD, pulmonary heart disease, bronchiectasis, tuberculosis and lung cancer, were not significantly different from those with COPD, perhaps due to the small sample size. Similarly, mood/state of mind and the results of the Gastroesophageal Reflux Disease Questionnaire (GERD-Q) were not significantly different between patients with COPD and those without. Red blood cell (RBC) counts, hemoglobin (HGB) levels, platelet (PLT) counts, and fibrinogen (FBG) levels in the peripheral blood were associated with COPD status, and the results revealed that RBC (p<0.001), HGB (p<0.001), PLT (p<0.02) and FBG (p<0.02) levels were also related to COPD (Table 3).

**Table 2:** Prevalence and GOLD grade distribution of COPD among adults, aged ≥20 years, in the Guizhou Province survey

	*n*	*%*
**COPD**		
Yes	518	9.60
No	4877	90.40
**GOLD of COPD**		
Grade 1	238	45.90
Grade 2	236	45.60
Grade 3	39	7.50
Grade 4	5	0.90

**Table 3:** Factors associated with COPD and Pre-COPD in adults, aged ≥20 years, Guizhou Province, 2012–2015 (N=5395)

*Factors*	*COPD* *(N=518)* *n (%)*			*p ^a^*	*Non-COPD* *(N=4877)* *n (%)*			*p ^b^*	*p ^c^*
	*Men* *279 (5.17)*	*Women* *239 (4.43)*	*Total* *518 (9.60)*	*0.0005*	*Men* *2199 (40.76)*	*Women* *2678 (49.64)*	*Total* *4877 (90.40)*	*0.39*	
**Age** (years)									
20–40	37 (41.57)	52 (58.43)	89	0.41	1035 (53.00)	918 (47.00)	1953	<0.0001	<0.0001
41–60	136 (60.44)	89 (39.56)	225	<0.0001	904 (40.65)	1320 (59.35)	2224	<0.0001	0.39
≥61	106 (51.96)	98 (48.39)	204	0.09	245 (37.98)	400 (62.02)	645	0.0001	<0.0001
**Education level**									
Primary school or lower	97 (45.75)	115 (54.25)	212	0.96	323 (28.43)	813 (71.57%)	1136	<0.0001	<0.0001
Junior/senior high school or technical secondary school	150 (58.82)	105 (41.18)	255	<0.0001	1326 (46.76)	1510 (53.24)	2836	0.48	0.0004
University/ junior college or research	32 (62.75)	19 (37.25)	51	0.02	550 (60.77)	355 (39.23)	905	<0.0001	<0.0001
**Body mass index** (kg/m^2^)									
<18.5 (underweight)	19 (55.88)	15 (44.12)	34	0.25	130 (48.69)	137 (51.31)	267	0.38	0.35
18.5–23.9 (normal weight)	142 (48.97)	148 (51.03)	290	0.31	1145 (43.05)	1515 (56.95)	2660	0.01	0.57
≥24.0 (overweight)	114 (60.32)	75 (39.68)	189	<0.0001	903 (47.15)	1012 (52.85)	1915	0.36	0.26
**Smoking status**									
Never smoker	66 (23.07)	220 (76.93)	286	<0.0001	807 (23.84)	2578 (76.16)	3385	<0.0001	<0.0001
Current smoker	186 (92.07)	16 (7.93)	202	<0.0001	1255 (93.37)	89 (6.63)	1344	<0.0001	<0.0001
Quit	27 (90.00)	3 (10.00)	30	<0.0001	137 (92.57)	11 (7.43)	148	<0.0001	0.003
Mother smoked in pregnancy	23 (69.70)	10 (30.30)	33	0.0063	83 (43.01)	110 (56.99)	193	0.42	0.02
Mother exposed to SHS in pregnancy	120 (55.30)	97 (44.70)	217	0.0067	924 (47.36)	1027 (52.64)	1951	0.28	0.45
**Biofuel exposure**									
Yes	5 (33.33)	10 (66.67)	15	0.33	78 (48.75)	82 (51.25)	160	0.48	0.67
Fry/stir-fry/roast	192 (55.01)	157 (45.99)	349	0.0009	1524 (46.63)	1744 (53.37)	3268	0.52	0.88
Coal >6 months	97 (48.50)	103 (51.50)	200	0.47	568 (42.17)	779 (57.83)	1347	0.01	<0.0001
LPG >6 months	53 (53.00)	47 (47.00)	100	0.16	433 (43.91)	553 (56.09)	986	0.24	0.65
Gas >6 months	38 (53.52)	33 (46.48)	71	0.20	484 (51.88)	449 (48.12)	933	0.0008	0.01
Grass >6 months	5 (33.33)	10 (66.67)	15	0.33	80 (49.69)	81 (50.31)	161	0.35	0.65
**Kitchen separate**									
Yes	259 (54.64)	215 (45.36)	474	0.0002	2050 (45.05)	2500 (54.95)	4550	0.38	0.17
No	19 (43.18)	25 (56.82)	44	0.72	149 (45.57)	178 (54.43)	327	0.90	0.17
**Medical history**									
Asthma	6 (50.00)	6 (50.00)	12	0.78	35 (45.45)	42 (54.55)	77	0.93	0.26
Chronic bronchitis	8 (42.11)	11 (57.89)	19	0.74	86 (43.65)	111 (56.35)	197	0.53	0.71
Emphysema COPD	4 (36.36)	7 (63.64)	11	0.52	34 (36.96)	58 (63.04)	92	0.09	0.73
Pulmonary heart disease	4 (66.67)	2 (33.33)	6	0.31	15 (45.45)	18 (54.55)	33	0.96	0.28
Bronchiectasis	1 (50.00)	1 (50.00)	2	0.91	3 (50.00)	3 (50.00)	6	0.84	0.21
Lung TB	7 (77.78)	2 (22.22)	9	0.06	29 (50.88)	28 (49.12)	57	0.54	0.32
Lung CA	3 (75.00)	1 (25.00)	4	0.24	26 (42.62)	35 (57.38)	61	0.61	0.38
**Mood/state of mind,** mean (95% CI) [adjusted p-values^d^]									
Peaceful	2.01 (1.88–2.14)	1.96 (1.81–2.11)	514	0.65	2.03 (1.98–2.08)	2.05 (2.01–2.09)	4829	0.61	0.30
Energetic	2.13 (2.00–2.26)	2.14 (1.99–2.29)	513	0.92	2.11 (2.06–2.16)	2.18 (2.13–2.22)	4838	0.06	0.83
Depressed	5.19 (5.07–5.31)	5.23 (5.09–5.37)	513	0.69	5.21 (5.16–5.25)	5.18 (5.14–5.23)	4839	0.48	0.77
Lethargic	4.21 (4.06–4.35)	4.3 (4.15–4.44)	514	0.39	4.38 (4.33–4.42)	4.33 (4.29–4.38)	4814	0.18	0.05
Nervous	3.68 (3.61–3.76)	3.66 (3.58–3.74)	513	0.70	3.64 (3.62–3.67)	3.58 (3.55–3.60)	4848	0.0007	0.04
Still enjoy things	1.35 (1.26–1.44)	1.34 (1.25–1.43)	513	0.91	1.38 (1.34–1.41)	1.38 (1.35–1.41)	4839	0.80	0.36
Afraid	3.69 (3.61–3.76)	3.64 (3.55–3.73)	513	0.45	3.69 (3.66–3.71)	3.62 (3.60–3.65)	4845	0.001	0.71
Can still laugh	1.45 (1.35–1.56)	1.38 (1.28–1.48)	513	0.30	1.34 (1.31–1.37)	1.39 (1.36–1.42)	4845	0.02	0.16
Worried	3.53 (3.43–3.62)	3.58 (3.49–3.67)	511	0.44	3.58 (3.55–3.61)	3.54 (3.51–3.57)	4845	0.08	0.91
Happy	3.52 (3.42–3.62)	3.59 (3.48–3.69)	513	0.37	3.61 (3.57–3.64)	3.61 (3.58–3.64)	4850	0.99	0.15
Relaxed	1.61 (1.51–1.71)	1.56 (1.46–1.66)	513	0.48	1.55 (1.52–1.58)	1.59 (1.56–1.62)	4846	0.10	0.68
Dullness	3.52 (3.43–3.61)	3.66 (3.58–3.74)	513	0.02	3.6 (3.57–3.63)	3.53 (3.50–3.56)	4844	0.0008	0.51
Frightened	1.34 (1.27–1.42)	1.35 (1.26–1.44)	513	0.88	1.29 (1.27–1.32)	1.36 (1.33–1.38)	4841	0.0005	0.55
Don’t care about appearance	3.34 (3.22–3.46)	3.43 (3.31–3.56)	512	0.31	3.45 (3.41–3.49)	3.47 (3.44–3.51)	4843	0.39	0.08
Tired	3.44 (3.33–3.55)	3.44 (3.32–3.56)	513	0.96	3.41 (3.37–3.44)	3.39 (3.35–3.43)	4846	0.57	0.34
Enthusiasm	1.37 (1.28–1.45)	1.38 (1.28–1.48)	512	0.86	1.37 (1.33–1.40)	1.36 (1.34–1.39)	4847	0.92	0.85
Panic	3.78 (3.72–3.84)	3.75 (3.68–3.82)	512	0.52	3.78 (3.76–3.80)	3.72 (3.70–3.74)	4850	0.0002	0.47
**GERD Q,** mean (95% CI) [adjusted p-values^d^]								
Heartburn	1.10 (1.05–1.15)	1.10 (1.05–1.15)	518	0.99	1.07 (1.06–1.09)	1.09 (1.08–1.11)	4866	0.07	0.22
Reflux	1.11 (1.06–1.15)	1.08 (1.03–1.13)	518	0.40	1.09 (1.08–1.11)	1.10 (1.09–1.12)	4864	0.44	0.92
Mid abdominal pain	1.09 (1.04–1.13)	1.07 (1.03–1.11)	518	0.56	1.07 (1.05–1.08)	1.09 (1.07–1.10)	4864	0.04	0.89
Nausea	1.04 (1.02–1.07)	1.05 (1.01–1.09)	518	0.77	1.07 (1.06–1.09)	1.07 (1.06–1.08)	4866	0.69	0.11
Less sleep	1.04 (1.01–1.07)	1.05 (1.02–1.09)	518	0.45	1.06 (1.04–1.07)	1.06 (1.04–1.07)	4868	0.85	0.39
Drug for reflux	1.01 (0.99–1.03)	1.04 (1.00–1.08)	518	0.12	1.03 (1.02–1.05)	1.03 (1.02–1.04)	4867	0.74	0.48
**Blood environment,** mean (95% CI) [adjusted p-values^d^]									
WBC	6.54 (6.30–6.77)	6.23 (5.99–6.48)	495	0.08	6.50 (6.43–6.58)	5.96 (5.9–6.02)	4616	<0.0001	0.02
NEU rate	59.61 (58.35–60.86)	60.21 (58.77–61.64)	497	0.53	58.74 (58.28–59.20)	59.7 (59.3–60.11)	4614	0.002	0.218
EOS rate	2.64 (2.35–2.94)	2.55 (2.19–2.92)	476	0.70	2.40 (2.30–2.50)	2.11 (2.02–2.20)	4467	<0.0001	0.0010
RBC	4.96 (4.90–5.03)	4.57 (4.49–4.65)	497	<0.0001	5.22 (5.19–5.24)	4.59 (4.57–4.61)	4616	<0.0001	0.001
HGB	154.12 (152.02–156.21)	135.62 (133.25–137.99)	497	<0.0001	160.26 (159.62–160.89)	136.5 (135.95–137.05)	4616	<0.0001	0.08
PLT	173.56 (166.81–180.31)	185.66 (177.46–193.87)	496	0.02	187.44 (185.08–189.79)	197.37 (194.93–199.81)	4608	<0.0001	<0.0001
FBG	5.47 (5.29–5.64)	5.18 (5.03–5.33)	476	0.02	5.40 (5.35–5.46)	5.29 (5.24–5.35)	4504	0.004	0.87
TC	1.76 (1.59–1.94)	1.67 (1.52–1.81)	474	0.41	1.84 (1.78–1.90)	1.62 (1.58–1.66)	4491	<0.0001	0.98
TCH	5.10 (4.98–5.23)	5.07 (4.92–5.22)	478	0.72	4.89 (4.84–4.94)	4.96 (4.91–5.00)	4518	0.04	0.002

a p for difference: a chi-squared test was conducted to evaluate the discrepancy in the distribution of men and women among COPD patients, with the FDR-adjusted p value presented.

b p for difference: a chi-squared test was conducted to evaluate the discrepancy in the distribution of men and women among the non-COPD population, with the FDR-adjusted p value presented.

c p for difference: a chi-squared test was conducted to evaluate the discrepancy between the distributions of the COPD and non-COPD populations, with the FDR-adjusted p value presented.

d Student’s t-test of differences.

## DISCUSSION

COPD is the fourth leading cause of death globally and the third leading cause of mortality in China^1,6^. It is caused by dynamic, cumulative, and repeated interactions between genes and the environment throughout a person’s lifetime, which can harm the lungs and disrupt their normal development and aging processes^12^. GOLD 2001 proposed the concept of GOLD stage 0, which means that patients have high-risk factors for COPD and persistent respiratory symptoms, but there is no airflow limitation as measured by the pneumometer (FEV1/FVC>0.7 after inhalation of bronchodilators), and it emphasizes that patients in GOLD stage 0 are at high risk of developing COPD in the future^7,13,14^. Since COPD begins in the early stages of life and it takes a long time for clinical symptoms to appear, GOLD 2024 suggests that early-stage COPD is defined as the biological early stage of the disease course^15^. ‘Biological early stage’ usually refers to the early stage of disease development. At this time, patients may not have obvious clinical symptoms yet, but there is already a certain degree of damage to the lungs and abnormal lung function trajectories (abnormal lung development and rapid decline in lung function). The early stage of life is a crucial period for lung growth and development. Early life risk factors such as intrauterine tobacco exposure, low birthweight and premature birth, severe respiratory tract infections in childhood, pneumonia or bronchitis, secondhand smoke exposure, and family poverty are closely related to the onset of chronic obstructive pulmonary disease in adulthood^8,16^.

Our research has found that smoking, including active or passive smoking during pregnancy, coal-based biofuels, cooking methods such as frying, stir-frying, and grilling, indoor smoke exposure, and low level of education etc., are all associated factors for COPD and Pre-COPD. Consistent with the literature reports^17-19^. Liu et al.^20^ reported that just three days of exposure to kitchen cooking fumes can cause pneumonia and damage the intestines, and seven days of continuous exposure is even more severe. Therefore, this research underscores the influence of environmental and lifestyle factors on the development of COPD and Pre-COPD.

With a rapidly aging population and heavy household and outdoor air pollutants, crowding, poor nutrition, infections, or other factors related to low socioeconomic status, the prevalence of COPD is expected to continue to rise^9^. The term ‘pre-disease stage’ refers to an intermediate state between health and disease, in which certain pathological biological mechanisms involved in disease progression have already been activated, but the possibility of returning to normal still exists^21^. Hence, terms such as ‘early COPD’, ‘mild COPD’, ‘young COPD’, and ‘Pre-COPD’ in the Global Initiative for Chronic Obstructive Lung Disease 2023 Report were proposed^9,16^. However, at present, research reports on ‘early COPD’, ‘mild COPD’, ‘young COPD’, and ‘Pre-COPD’ are rare. Martinez et al.^22^ highlighted the need to distinguish ‘early disease’ from late ‘mild disease’, proposed an operational definition of early COPD for use in research studies, and attempted to unify current views on potential disease mechanisms. The term ‘Pre-COPD’ describes a diverse set of conditions in which certain characteristics of COPD are evident, yet the defining characteristic of COPD – persistent chronic airflow obstruction – is absent^7,23^. Çolak et al.^24^ discussed the occurrence of COPD during follow-up in individuals who did not display airflow obstruction, defined as FEV1/FVC<0.7, yet have any of the following conditions, which they classify as a pre-disease state of COPD: 1) symptoms of chronic bronchitis; 2) PRISm (preserved ratio impaired spirometry with FEV1/FVC ≥70% and FEV1 <80% predicted); 3) ‘early airflow limitation’ with FEV1/FVC >70% but <LLN (Lower Limit of Normal); or 4) asthma. According to the criteria proposed by Çolak et al.^24^, there were 1264 adult subjects considered to have Pre-COPD, accounting for 8.92% of the total study subjects; but the age-standardized prevalence of Pre-COPD was 7.20% (95% CI: 5.9–8.8) in the China Pulmonary Health study. Although we found no significant difference in mood among those with COPD, previous research has showed that preserved ratio impaired spirometry (PRISm) and airflow obstruction carry equally high risks of depression and anxiety. PRISm recognition may contribute to the prevention of depression and anxiety^25^. Thus, inconsistent with previous reports, we attribute this discrepancy to our limited sample size. Wang et al.^26^ conducted a survey among Chinese people aged >20 years and found that the age-standardized prevalences of PRISm, Pre-COPD, young COPD, and mild COPD approximately were 5.5%, 7.2%, 1.1%, and 3.1%, respectively. For example, only a small proportion of PRISm and Pre-COPD individuals would certainly progress to affirmed COPD in the coming years^27,28^. A history of intrauterine tobacco exposure, hospitalization for bronchitis or pneumonia in childhood, frequent cough symptoms, and tobacco smoke exposure were high-risk factors for the future development of COPD in young people and Pre-COPD. Agustí et al.^29^ reported that approximately a quarter of patients diagnosed and treated with COPD in real life do not fulfil the COPD definition of non-fully reversible airflow limitation, and can be classified instead as having either normal spirometry (Pre-COPD) or a PRISm. Regardless of how they are conceptually considered, one key issue to be resolved is how individuals with Pre-COPD/early COPD will be detected to facilitate the implementation of preventive measures^30^. Hence, awareness of risk factors is essential for the development of public health policy and rational planning of healthcare resources and contributes to the effective self-management of COPD and Pre-COPD patients. Combining our results, increasing importance should be given to screening and health education on COPD and Pre-COPD in the general population, and lung function tests for people at high risk of COPD for early diagnosis and treatment of the disease to reduce the social and economic burden caused by COPD.

### Limitations

Our study has several limitations. First, we used province-level random sampling, yet coverage of remote villages was incomplete, and non-response may have introduced selection bias. Second, the cross-sectional design precludes causal inference, and residual confounding by unmeasured variables (e.g. indoor biomass exposure) cannot be excluded. Third, comparisons between COPD and Pre-COPD groups were unadjusted for multiple covariates, increasing the associated of false-positive findings. Fourth, people with asthma were not excluded from the study population, which might cause an overestimate of COPD prevalence in younger age groups. Finally, because the survey was conducted in a single Chinese province, generalizability to other countries or ethnic groups is limited.

Our findings reveal a concerningly high prevalence of COPD and Pre-COPD among the adult population in Guizhou, driven by a complex interplay of environmental, behavioral, and socio-economic factors. The identified associated factors, particularly those related to smoking and indoor air quality, highlight critical areas for public health intervention and policy reform. Addressing these factors through targeted strategies may prevent people with Pre-COPD from rapidly progressing to COPD, and which mitigate the burden of COPD in this population and contribute to improved respiratory health outcomes. Subsequently, we will continue to follow up the development of patients diagnosed with COPD and Pre-COPD, and further conduct subgroup analyses on the associated factors of COPD in subjects with normal pulmonary ventilation function but chronic bronchitis or small airway dysfunction.

## CONCLUSIONS

This analysis shows that the prevalence of COPD and Pre-COPD in Guizhou adults exceeds the national Chinese average. Smoking, including active or passive smoking during pregnancy, coal-based biofuels, cooking methods such as frying, stir-frying and grilling, indoor smoke exposure and low level of education, were identified to be higher in the COPD and pre-COPD population groups.

## Data Availability

The data supporting this research are available from the authors on reasonable request.

## References

[1] Wachami N, Guennouni M, Iderdar Y, et al. Estimating the global prevalence of chronic obstructive pulmonary disease (COPD): A systematic review and meta-analysis. BMC Public Health. 2024;24(1):297. doi:10.1186/s12889-024-17686-9 38273271 PMC10811845

[2] GBD Chronic Respiratory Disease Collaborators. Prevalence and attributable health burden of chronic respiratory diseases, 1990–2017: A systematic analysis for the Global Burden of Disease Study 2017. Lancet Respir Med. 2020;8(6):585-96. doi:10.1016/S2213-2600(20)30105-3 32526187 PMC7284317

[3] Say L, Chou D, Gemmill A, et al. Global causes of maternal death: A WHO systematic analysis. Lancet Glob Health. 2014;2(6):323-333. doi:10.1016/S2214-109X(14)70227-X 25103301

[4] Zhu B, Wang Y, Ming J, Chen W, Zhang L. Disease burden of COPD in China: A systematic review. Int J Chron Obstruct Pulmon Dis. 2018;13:1353–1364. doi:10.2147/COPD.S161555 29731623 PMC5927339

[5] Zhong N, Wang C, Yao W, et al. Prevalence of chronic obstructive pulmonary disease in China: A large, population-based survey. Am J Respir Crit Care Med. 2007;176(8):753-760. doi:10.1164/rccm.200612-1749OC 17575095

[6] Wang C, Xu J, Yang L, et al. Prevalence and risk factors for chronic obstructive pulmonary disease in China (the China Pulmonary Health [CPH] study): A national cross-sectional study. Lancet. 2018; 391(10131):1706-1717. doi:10.1016/S0140-6736(18)30841-9 29650248

[7] Han MK, Agusti A, Celli BR, et al. From GOLD 0 to Pre-COPD. Am J Respir Crit Care Med. 2021;203(4):414-423. doi:10.1164/rccm.202008-3328PP 33211970 PMC7885837

[8] Agustí A, Celli BR, Criner GJ, et al. Global initiative for chronic obstructive lung disease 2023 Report: GOLD executive summary. Eur Respir J. 2023;61(4):2300239. doi:10.1183/13993003.00239-2023 36858443 PMC10066569

[9] Agustí A, Celli BR, Criner GJ, et al. Global initiative for chronic obstructive lung disease 2023 report: GOLD executive summary. Am J Respir Crit Care Med. 2023;207(7):819-837. doi:10.1164/rccm.202301-0106PP 36856433 PMC10111975

[10] Stolz D, Mkorombindo T, Schumann DM, et al. Toward the elimination of chronic obstructive pulmonary disease: A Lancet commission. Lancet. 2022;400(10356):921-972. doi:10.1016/S0140-6736(22)01273-9 36075255 PMC11260396

[11] Labaki WW, Rosenberg SR. Chronic obstructive pulmonary disease. Ann Intern Med. 2020;173(3):17-32. doi:10.7326/AITC202008040 32745458

[12] Agusti A, DeMeo DL, Faner R. Pathogenesis of chronic obstructive pulmonary disease: Understanding the contributions of gene-environment interactions across the lifespan. Lancet Respir Med. 2022;10:512-524. doi:10.1016/S2213-2600(21)00555-5 35427533 PMC11428195

[13] Pauwels RA, Buist AS, Calverley PM, Jenkins CR, Hurd SS, GOLD Scientific Committee. Global strategy for the diagnosis, management, and prevention of chronic obstructive pulmonary disease. NHLBI/WHO Global Initiative for Chronic Obstructive Lung Disease (GOLD) Workshop summary. Am J Respir Crit Care Med. 2001;163(5):1256-1276. doi:10.1164/ajrccm.163.5.2101039 11316667

[14] Venkatesan P. GOLD COPD report: 2024 update. Lancet Respir Med. 2024;12(1):15-16. doi:10.1016/S2213-2600(23)00461-7 38061380

[15] Luo P, He J, Wan X, et al. Association between birth weight and chronic obstructive pulmonary disease in the UK Biobank: A prospective cohort study. BMJ Open Respir Res. 2024;11(1):e002366. doi:10.1136/bmjresp-2024-002366 PMC11647385 39668106

[16] Wu F, Deng ZS, Tian HS, Li HQ, Zhou YM. Zhonghua Jie He He Hu Xi Za Zhi. 2023;46(10):1028-1034. doi:10.3760/cma.j.cn112147-20230223-00085 37752048

[17] Oswald NC, Medvei VC. Chronic bronchitis: The effect of cigarette smoking. Lancet. 1955;269(6895):843-844. doi:10.1016/s0140-6736(55)93480-2 13264626

[18] Sana A, Somda SMA, Meda N, Bouland C. Chronic obstructive pulmonary disease associated with biomass fuel use in women: A systematic review and meta-analysis. BMJ Open Respir Res. 2018;5(1):000246. doi:10.1136/bmjresp-2017-000246 PMC5786909 29387422

[19] Mortimer K, Montes de Oca M, Salvi S, et al. Household air pollution and COPD: Cause and effect or confounding by other aspects of Poverty. Int J Tuberc Lung Dis. 2022;26(3):206-216. doi:10.5588/ijtld.21.0570 35197160 PMC8886958

[20] Bailiang Liu, Ge Wang, Lina Wang, et al. Unraveling cross-organ impacts of airborne pollutants: A multiomics study on respiratory exposure and gastrointestinal health. Environ Sci Technol. 2024;58(35): 15511-15521. doi:10.1021/acs.est.4c06035 39145585

[21] Roche N, Han MK. The Evolving Contours of Chronic Obstructive Pulmonary Disease. Am J Respir Crit Care Med. 2024;210(5):535-537. doi:10.1164/rccm.202403-0565ED 38564415 PMC11389576

[22] Martinez FJ, Han MK, Allinson JP, et al. At the root: Defining and halting progression of early chronic obstructive pulmonary disease. Am J Respir Crit Care Med. 2018;197(12):1540-1551.doi:10.1164/rccm.201710-2028PP 29406779 PMC6006401

[23] Spittle DA, Thomas M, Stevens C, et al. Symptoms of COPD in the absence of airflow obstruction are more indicative of pre-COPD than overdiagnosis. ERJ Open Res. 2024;10(5):00264-2024. doi:10.1183/23120541.00264-2024 39351384 PMC11440379

[24] Çolak Y, Lange P, Vestbo J, et al. Susceptible young adults and development of COPD later in life. Am J Respir Crit Care Med. 2024;210(5):607-617. doi:10.1164/rccm.202308-1452OC 38364200

[25] Yang K, Wang L, Shen J, Chen S, Liu Y, Chen R. The association between preserved ratio impaired spirometry and adverse outcomes of depression and anxiety: Evidence from the UK Biobank. Psychol Med. doi:10.1017/S0033291724002162 PMC11496235 39324390

[26] Lei J, Huang K, Wu S, et al. Heterogeneities and impact profiles of early chronic obstructive pulmonary disease status: Findings from the China Pulmonary Health Study. Lancet Reg Health West Pac. 2024;45:101021. doi:10.1016/j.lanwpc.2024.101021 38352242 PMC10862401

[27] Wijnant SRA, De Roos E, Kavousi M, et al. Trajectory and mortality of preserved ratio impaired spirometry: The Rotterdam Study. Eur Respir J. 2020;55(1):1901217. doi:10.1183/13993003.01217-2019 31601717

[28] Divo MJ, Liu C, Polverino F, et al. From pre-COPD to COPD: A simple, low cost and easy to IMplement (SLIM) risk calculator. Eur Respir J. 2023;62(4): 2350806. doi: 10.1183/13993003.50806-2023 37678951 PMC10533946

[29] Agustí A, Hughes R, Rapsomaki E, et al. The many faces of COPD in real life: A longitudinal analysis of the NOVELTY cohort. ERJ Open Res. 2024;10(1):00895-2023. doi:10.1183/23120541.00895-2023 38348246 PMC10860203

[30] Lin CH, Cheng SL, Chen CZ, Chen CH, Lin SH, Wang HC. Current progress of COPD early detection: Key points and novel strategies. Int J Chron Obstruct Pulmon Dis. 2023;18:1511-1524. doi:10.2147/COPD.S413969 37489241 PMC10363346

